# Potential of uPAR, αvβ6 Integrin, and Tissue Factor as Targets for Molecular Imaging of Oral Squamous Cell Carcinoma: Evaluation of Nine Targets in Primary Tumors and Metastases by Immunohistochemistry

**DOI:** 10.3390/ijms24043853

**Published:** 2023-02-14

**Authors:** Mads Lawaetz, Anders Christensen, Karina Juhl, Kirstine Karnov, Giedrius Lelkaitis, Anne-Marie Kanstrup Fiehn, Andreas Kjaer, Christian von Buchwald

**Affiliations:** 1 Department of Otolaryngology, Head and Neck Surgery and Audiology, Rigshospitalet, Copenhagen University Hospital, 2100 Copenhagen, Denmark; 2 Department of Clinical Physiology, Nuclear Medicine and PET and Cluster for Molecular Imaging, Copenhagen University Hospital-Rigshospitalet & Department of Biomedical Sciences, University of Copenhagen, 2100 Copenhagen, Denmark; 3 Department of Pathology, Rigshospitalet, Copenhagen University Hospital, 2100 Copenhagen, Denmark; 4 Department of Clinical Medicine, University of Copenhagen, 2200 Copenhagen, Denmark

**Keywords:** oral squamous cell carcinoma, lymph node metastases, molecular imaging, immunohistochemistry, urokinase-type plasminogen activator receptor, tissue factor, integrin αvβ6

## Abstract

No clinically approved tumor-specific imaging agents for head and neck cancer are currently available. The identification of biomarkers with a high and homogenous expression in tumor tissue and minimal expression in normal tissue is essential for the development of new molecular imaging targets in head and neck cancer. We investigated the expression of nine imaging targets in both primary tumor and matched metastatic tissue of 41 patients with oral squamous cell carcinoma (OSCC) to assess their potential as targets for molecular imaging. The intensity, proportion, and homogeneity in the tumor and the reaction in neighboring non-cancerous tissue was scored. The intensity and proportion were multiplied to obtain a total immunohistochemical (IHC) score ranging from 0–12. The mean intensity in the tumor tissue and normal epithelium were compared. The expression rate was high for the urokinase-type plasminogen activator receptor (uPAR) (97%), integrin αvβ6 (97%), and tissue factor (86%) with a median total immunostaining score (interquartile range) for primary tumors of 6 (6–9), 12 (12–12), and 6 (2.5–7.5), respectively. For the uPAR and tissue factor, the mean staining intensity score was significantly higher in tumors compared to normal epithelium. The uPAR, integrin αvβ6, and tissue factor are promising imaging targets for OSCC primary tumors, lymph node metastases, and recurrences.

## 1. Introduction

Despite advances in diagnostic techniques and postoperative treatment, poor survival and high recurrence rate remain for patients with oral squamous cell carcinoma (OSCC) [1]. The primary curative treatment is surgery, where the adequate resection margins (>5 mm) are one of the most important prognosticators [2,3]. Achieving radical resection margins is challenging when the tumor is surrounded by multiple functionally and aesthetically critical structures and the border between the tumor and normal tissue is not clearly delineated. This is reflected in a positive margin rate of 12–30% for OSCC, one of the highest rates among all solid tumors [2,4,5]. Additionally, the detection and removal of regional lymph node metastases by neck dissection is a challenge due to a significant risk of occult microscopic disease that is not detected by conventional preoperative imaging [6]. Currently, there are no established real-time intraoperative imaging techniques for distinguishing healthy tissue from tumor tissue in OSCC. The surgeons rely on preoperative imaging and intraoperative visual and tactile information. Intraoperative margin assessment may be performed by use of frozen section microscopy, which is time-consuming and prone to sampling and interpretation errors [7].

Molecular imaging is a rapidly emerging field for the diagnosis and treatment of cancer, particularly head and neck cancer, in which several targets and modalities have been studied and are under development [8]. Due to advancements in imaging hardware and fluorophore biochemistry, targeted fluorescence guided surgery (FGS) is one of the most promising real-time intraoperative imaging techniques. Especially fluorophores with excitation and emission in the near-infrared (NIR) spectrum, such as indocyanine green (ICG) and IRDye800CW, have been investigated due to a relatively high penetration depth compared to other wavelengths [9,10]. Despite intensive research, no clinically approved tumor-specific imaging agents for head and neck cancer surgery are currently available [11]. The identification of biomarkers with a high and homogenous expression in tumor tissue and minimal expression in normal tissue is essential for the development of new molecular imaging targets in head and neck cancer.

The vascular endothelial growth factor receptor 1 and 2 (VEGFR1 and VEGFR2) play important roles in tumor angiogenesis [12]. A high expression of both receptors has been reported in OSCC [13] and several studies have investigated these receptors as targets for molecular imaging in different cancers [14]. Integrin αvβ3 is another receptor expressed by tumor cells that plays an important role in tumor angiogenesis [15] and molecular imaging, and has been explored in several different cancers with promising results [16]. Integrin αvβ6 is a member of the same family that has been more thoroughly studied [17,18,19]. Integrin αvβ6 is important for cell migration as it facilitates cell-to-cell and cell-to-extracellular matrix adhesion. In OSCC, integrin αvβ6 has been found to be upregulated, especially at the invasive margin [20], and involved in different hallmarks of cancer including epithelial to mesenchymal transition [21], invasion, and migration [20,22]. The epithelial cell adhesion molecule (EpCAM), like integrins, is a cell adhesion receptor implicated in metastasis. It has been identified as being overexpressed in several malignancies, including OSCC [23], and several studies have already investigated the use of both fluorescence and radionuclide probes [24,25]. Cathepsin E and Poly(ADP-ribose)polymerase-1 (PARP-1) are both intracellular enzymes that have been shown to be overexpressed in a variety of malignancies [26,27]. PARP-1 has been examined as a PET-imaging target and a target for fluorescence imaging in OSCC [28,29,30], whereas Cathepsin E expression in OSCC has not been previously described. However, a fluorescence probe has been developed for Cathepsin E and tested in vivo [31]. The urokinase-type plasminogen activator receptor (uPAR) is a GPI-anchored cell membrane receptor that turns plasminogen into plasmin at the cell surface, thus degrading the extracellular matrix [32]. uPAR has been found to be upregulated in most solid cancers where it facilitates cell invasion and metastasis, and a high expression has been associated with poor prognosis and metastases [33]. The tissue factor, a transmembrane glycoprotein that stimulates the extrinsic coagulation pathway, is thought to have a significant role in tumor progression [34]. An overexpression of the tissue factor has been reported in several malignancies and is related with poor clinical outcomes [35,36].

Our aim was to investigate the immunohistochemical (IHC) expression of the above mentioned, nine interesting imaging targets in both primary tumor and matched metastatic tissue from OSCC to assess their potential as targets for molecular imaging. For a subgroup, the tissue from recurrent disease was evaluated.

## 2. Results

### 2.1. Patient Characteristics

In this population of 41 patients with OSCC, the median age at diagnosis was 58 years (range 23–81 years), and 26 (63%) of the patients were male (Table 1). The majority of tumors (73%) were moderately differentiated, and tumors were located in the floor of mouth (56%) and oral tongue (44%). All pathologic T-stages were represented. The majority of tumors were in stage T1 or T2 at the time of surgery and 38 patients (93%) had histological confirmed lymph node metastases. Surgery aiming radicality was the first line of treatment for all patients.

### 2.2. Immunohistochemical Staining

**Figure 1:**
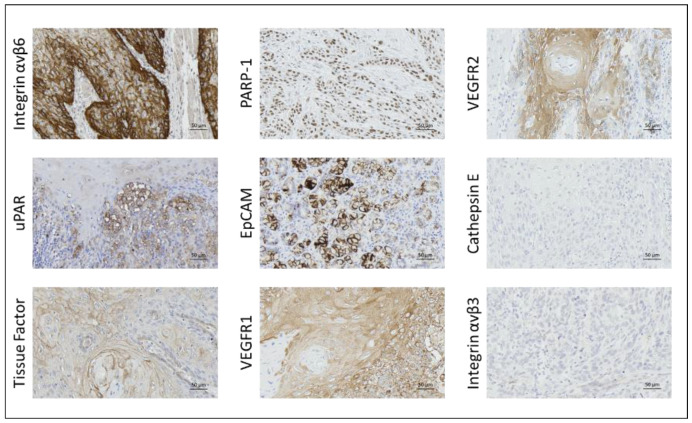
Expression of integrin αvβ6, tissue factor, PARP-1, uPAR, VEGFR1, EpCAM, VEGFR2, Cathepsin E and integrin αvβ3 in primary tumor tissue of OSCC.

**Figure 2:**
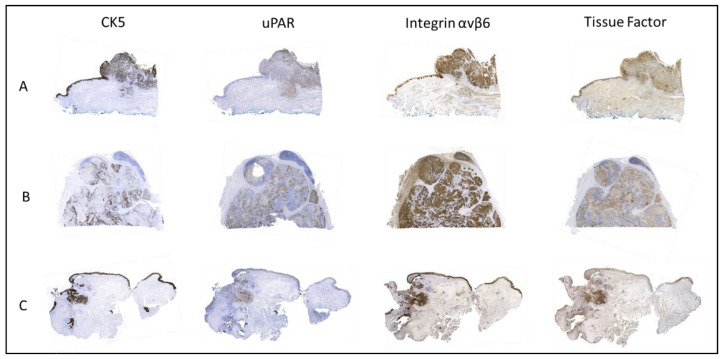
Expression of CK5 (for visualization of tumor localization), uPAR, integrin αvβ6 and tissue factor in (**A**) primary tumor, (**B**) lymph node metastases, and (**C**) local recurrence.

**Figure 3:**
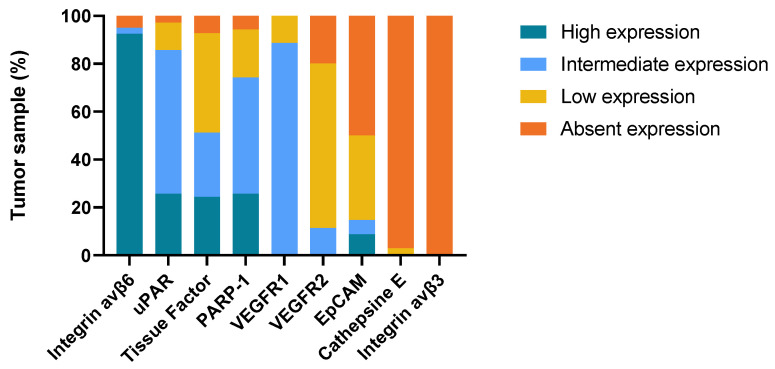
Expression of imaging targets in OSCC primary tumors.

**Figure 4:**
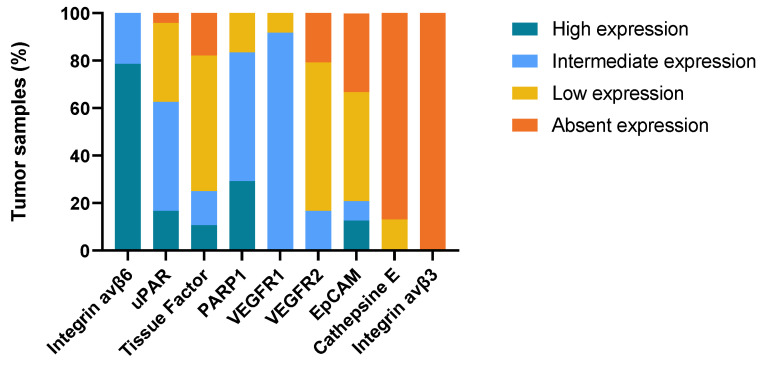
Expression of imaging targets in OSCC lymph node metastases.

Primary tumor tissue was obtained from all 41 patients. In a number of patients, there was insufficient remaining tumor tissue to perform IHC staining for all nine targets, and normal mucosa was not present or only present in some sections. Formalin-fixed, paraffin-embedded (FFPE) blocks containing metastatic tissue were available for 28 patients, while local recurrence tissue was obtained from eight patients. A representative image for each target’s immunohistochemical staining is shown in Figure 1 and the three most promising biomarkers in matched tumor samples from the same patient is shown in Figure 2. The intensity, proportion, and total immune staining score for all targets are shown in Table 2. An overview of the final expression category in primary tumors and metastases of all biomarkers is illustrated in Figure 3 and Figure 4, respectively.

#### 2.2.1. Integrin αvβ6

Integrin αvβ6 expression was seen in nearly all tumor samples (97%) with strong membrane and cytoplasmic staining in most tumor cells. There was a distinct demarcation between tumor cells and immune cells in lamina propria and surrounding tissue in submucosa. The staining was homogenous in 80% of all tumor samples (Table 3). The median staining scores (interquartile range) for primary tumor, lymph node metastases, and local tumor recurrence were 12 (12–12), 12 (9.75–12), and 12 (12–12), respectively. Except for a weak staining of muscle cells and a moderate staining of salivary gland ducts, no other normal cells in the subepithelial layers were positive. Integrin αvβ6 was also expressed in normal epithelium.

#### 2.2.2. uPAR

The overall expression rate was 97% with highly tumor-specific staining, which was rated as homogeneous in 51% of the samples. uPAR was expressed in 23/24 metastases (96%). Both membrane and cytoplasmic staining were found in tumor cells. The total immune staining scores for primary tumor cells, lymph node metastases, and local tumor recurrence tissue were 6 (6–9), 6 (4–8), and 6 (6–9.75), respectively. Normal epithelium exhibited no staining, except for in four cases where weak epithelial staining was seen. In one case, moderate staining of a lichen planus lesion was observed in the periphery of the tumor. There was a clear contrast between tumor and surrounding tissue at the deep tumor margin. Weak to moderate staining was observed in granulocytes.

#### 2.2.3. Tissue Factor

The overall expression rate of tissue factor in tumor tissue was high (86%), but only with a homogenous pattern in 3% of tumor samples. In half of the primary tumor samples, tissue factor showed moderate to intense expression. In lymph node metastases, expression was mainly weak and moderate. Staining scores for primary tumor cells, lymph node metastases, and local recurrence tumor tissue were 6 (2.5–7.5), 2 (1–5.5), and 4 (0–6), respectively. Normal epithelium expressed tissue factor in approximately 80% of the samples, although the staining in this compartment was mostly weak. Salivary duct and acini cells also showed a weak expression of tissue factor.

#### 2.2.4. PARP-1

A high overall expression rate was seen for PARP-1 (97%), with positive staining of tumor nuclei, albeit heterogeneously. For primary tumor cells, lymph node metastases, and local recurrence tumor tissue, the staining scores were 6 (4–9), 6 (6–9), and 6 (4–8), respectively. Nevertheless, the staining was not very tumor-specific, as several normal cells were also stained. Lymphocytes, endothelium, muscle tissues, nerve fibers, salivary gland tissues, plasma cells, and normal epithelium exhibited variable nuclei staining.

#### 2.2.5. VEGFR1

All tumors were positive for VEGFR1, but the staining was not tumor-specific and contrasted poorly with the normal stroma and epithelium. The VEGFR1 staining scores for primary tumor, lymph node metastases, and recurrent tumor tissue were 8 (6–8), 8 (6.5–8), and 8 (3–6), respectively. Macrophages, plasma cells, nerve fibers, endothelium, muscle tissues, and salivary gland tissues had expression of VEGFR.

#### 2.2.6. EpCAM

EpCAM was expressed in 57% of all tumor samples, but only 3% exhibited a homogenous pattern. In tumor cells, membrane and cytoplasmic stains were seen. The intensity of EpCAM positive tumors varied but was generally weak to moderate. Total IHC scores were 0.5 (0–2.5), 1.5 (0–3), and 1 (0–6) for primary tumor cells, lymph node metastases, and local recurrence tumor tissue, respectively. Rarely were EpCAM-positive macrophages and plasma cells observed. Normal epithelium exhibited no staining.

#### 2.2.7. VEGFR2

The overall expression rate of VEGFR2 was 79%, with no tumors displaying homogenous expression pattern. The VEGFR2 antibody staining was present in the cytoplasm of the tumor cells, although it was mainly weak. The staining scores for primary tumor tissue, lymph node metastases, and local recurrence were 2 (1–4), 2 (1–4), and 2 (0–2), respectively. Moderate to weak expression was also seen in normal oral squamous epithelium in 29% of samples. No expression was seen in the stroma surrounding tumor.

#### 2.2.8. Cathepsin E and Integrin αvβ3

Only one primary tumor and three lymph node metastases showed Cathepsin E expression. No expression of integrin αvβ3 was observed in primary tumors, metastases, or tissue from local recurrence. The staining scores for both biomarkers for primary tumor cells, lymph node metastases, and local recurrence tumor tissue were 0 (0–0), 0 (0–0), and 0 (0–0).

### 2.3. Intensity of Staining in Normal Oral Mucosal Epithelium vs. Tumor Tissue

The mean staining intensity score between normal epithelium and tumor tissue was compared for all samples where both components were present. The staining intensity was significantly higher in tumors compared to normal epithelium in uPAR (*p* < 0.001, n = 37), VEGFR2 (*p* = 0.002, n = 41), VEGFR1 (*p* = 0.001, n = 41), PARP-1 (*p* = 0.003, n = 40), and tissue factor (*p* < 0.001, n = 47). No difference in staining intensity between tumor tissue and normal epithelium was seen for integrin αvβ6 (*p* = 0.380, n = 47) or EPCAM (*p* = 0.130, n = 39).

### 2.4. Biomarker Expression in Primary Tumor Compared to Lymph Node Metastases and Tissue from Local Recurrence (T-Site)

We examined the correlation between total immune staining scores in primary tumors and lymph node metastases for each target in cases where tissue from both locations were available. We identified 28 primary cancers with accessible tissue from lymph node metastasis. All targets with tumor staining exhibited a positive Spearman rank correlation value. However, only uPAR (spearman correlation = 0.554, *p* = 0.014), tissue factor (spearman correlation = 0.615, *p* = 0.001), and VEGFR2 (Spearman correlation = 0.765, *p* < 0.001) had a significant positive correlation between total immune staining scores in primary tumor and lymph node metastases. Due to small numbers of cases with recurrence, no significant correlation was found between the total immune staining scores in primary tumors and tumor tissue from local recurrence, but a tendency toward positive correlation was seen for uPAR (spearman correlation = 0.395; *p* = 0.510), EpCAM (spearman correlation = 0.111, *p* = 0.834), and PARP-1 (spearman correlation = 0.064, *p* = 0.905)

## 3. Discussion

In this study, we have evaluated nine potential molecular imaging targets from 41 OSCC patients with tissue samples from the primary tumor, lymph node metastases, and local recurrence. This is, to the best of our knowledge, the first study to investigate and compare multiple potential targets in both primary OSCC tumors and their metastases. Based on immunohistochemical expression levels and expression patterns in the tumor, normal epithelium, and surrounding tissue, it was revealed that the uPAR, integrin αvβ6, and tissue factor represent attractive molecular imaging targets in OSCC due to a high overall expression rate of 97%, 97%, and 86%, respectively. The high expression rates of uPAR (96%) and integrin αvβ6 (100%) in lymph node metastases indicate a potential in FGS for detecting lymph node metastases during sentinel lymph node biopsy or neck dissection, which could potentially spare healthy nodes.

We found a highly tumor-specific uPAR expression in most tumor samples (97%), with a moderate to intense staining in both primary tumors and metastases. Our results are in accordance with previous immunohistochemical studies that have also found a high tumor-specific expression of uPAR in OSCC, with an absence of staining in the surrounding normal squamous epithelium and weak expression in tumor-associated inflammatory cells (macrophages, neutrophils, and fibroblasts), with a sharp demarcation at the deep tumor margin [37,38,39]. Interestingly, our current study demonstrated uPAR expression in 96% of metastasis, which indicates that combined targeted strategies against the tumor as well as metastatic disease seem possible. Different molecular imaging modalities have been explored for uPAR. In clinical trials, uPAR-targeted PET imaging using a peptide-based tracer has been studied for several cancers including OSCC, where a prognostic value was demonstrated [40,41,42,43]. No studies have yet investigated the diagnostic potential of uPAR-targeted PET imaging in OSCC, but a Phase II clinical trial is currently underway (NCT02960724). Few clinical studies have been conducted on FGS using uPAR-directed probes. In a cell-line-based xenograph proof-of-concept study conducted at our institution, it was shown that uPAR-targeted optical near-infrared fluorescence imaging using ICG conjugated to AE-105 can be used to identify small lymph node metastases during surgery [44]. Boonstra et al. also investigated uPAR-targeted FGS in cell-line-based xenograph models with an antibody-based tracer (hybrid ATN 658) conjugated to a fluorophore (ZW800-1), and showed that this modality could also identify primary tumors and lymph node metastases [45]. Clinical trials investigating uPAR-targeted FGS are ongoing in patients with oral cancer, lung cancer, and glioblastoma (EudraCT no. 2022-001361-12, 2021-004389-37 and 2020-003089-38).

The tissue factor also demonstrated a tumor-specific expression, but at a lower rate (86%) and with a more heterogeneous pattern than uPAR. The expression of the tissue factor in lymph node metastases was less compared to the primary tumor tissue. These results are consistent with similar immunohistochemistry studies on primary tumor tissue from oral and oropharyngeal squamous cell carcinoma, which found tissue factor expression rates of 58% and 76%, respectively [37,46]. As, an imaging target tissue factor has been poorly investigated in OSCC, but the potential in several other cancers has been explored. In preclinical studies, the tissue factor has been investigated as a target for FGS, SPECT, and PET using tissue factor-specific monoclonal antibodies in both anaplastic thyroid cancer, glioblastoma, and pancreatic cancer xenografts with promising effect [47,48,49,50]. In 2021, an antibody drug (tisotumab vedotin)-targeting tissue factor was approved by FDA for treatment of metastatic cervical cancer [51]. Subsequently the tissue factor-targeted PET-imaging with a protein (FVIIa) labeled with ^18^F was successfully tested first in a human study and proposed as a future diagnostic tool prior to tissue factor-targeted treatment [52]. The high expression of the tissue factor in OSCC and the recent development of tissue factor-targeted tracers in other solid cancers makes it a promising imaging agent in OSCC.

Integrin αvβ6 was also highly expressed in our study, with a clear contrast at the deep tumor margin. However, a high integrin αvβ6 expression was also seen in normal squamous cell epithelium without a significant difference in the intensity score between a tumor and normal epithelium. Our findings suggest that molecular imaging drugs targeting integrin αvβ6 may provide a distinct contrast at the deep margin but less at the superficial margins. These results are in line with those obtained by Baart et al., who investigated the immunohistochemical expression of integrin αvβ6 in both OSCC and cutaneous squamous cell carcinoma of the head and neck [38]. They also proposed integrin αvβ6 as a target for FGS in OSCC, especially due to the clear discrimination at the deep margin and compared to EGFR, they found less staining of the normal epithelium. Integrin αvβ6 has been studied as a PET-imaging target in different cancers. In 2019, Hausner et al. successfully performed a first in human studies by exploring PET/CT with a radiolabeled integrin αvβ6-binding peptide in patients with metastatic colon, breast, and pancreas cancer [18]. Later, Quigley et al. tested a Ga-68-labeled peptide (Ga-68-Trivehexin) for human PET/CT imaging of head, neck, and pancreatic cancer, with results showing a high tumor-specific uptake and no uptake in tumor-associated inflammation [19]. Integrin αvβ6 has, to our knowledge, not been tested as a target for fluorescent imaging in OSCC patients. However, Ilyia et al. showed imaging potential in in vitro head and neck cancer models with quantum dots conjugated to an integrin αvβ6-specific peptide [53]. A human trial by de Valk et al. has studied integrin αvβ6-targeted near-infrared fluorescent peptides (cRGD-ZW800-1) in 12 patients with colon carcinoma and was able to show cancer-specific imaging in both open and laparoscopic surgery [54]. Studies investigating integrin αvβ6 as a target for fluorescent imaging in OSCC have not yet been published, but a clinical trial with cRGD-ZW800-1 (NCT 04191460) is planned to investigate whether this modality can improve the rate of adequate surgical resection margins in OSCC.

PARP-1 showed mostly moderate and moderate to high expression levels in the tumor nuclei, but it appears less suitable as an imaging target compared to uPAR, αvβ6, and tissue factor, owing to the non-specific staining of several different cell-types in the lamina propria and submucosa as well as the staining of normal squamous epithelium. Even though some expressions of PARP-1 are present in normal tissues, this biomarker might not be excluded as a target for molecular imaging, because the density of the nuclei in tumor cells are higher compared to normal tissues [28]. Kossatz et al. recently investigated a topically applied PARP-1-specific fluorescence agent for the use of early diagnosis of OSCC in a Phase 1 study with 12 patients, where the fluorescence signal showed a tumor to normal ratio > 3 [30]. However, the topical approach is probably confined to early stage disease or screening of mucosal lesions, as the penetration depth is limited (300 μm in the trial by Kossatz et al.). VEGFR1 and VEGFR2 did not appear promising for imaging purposes in our study, as their expression was limited, and the tumor specificity was low. No studies have yet examined the molecular imaging of these targets in OSCC, but different angiogenesis inhibitors for the treatment of head and neck squamous cell carcinoma have been thoroughly investigated, with bevacizumab being the most promising [55].

This study has some limitations. First, a biomarkers appropriateness as an imaging target is determined by several factors in addition to its overexpression. The target selection criteria system has been suggested as a tool to identify potential imaging targets and consists of seven different criterions. However, several of these are either difficult to measure (tumor to normal ratio greater than 10) or questionable (internalization of the tracer) [56]. Second, immunohistochemistry has several inherited limitations, including the selection of an antibody clone, which can affect the intensity and proportion of the stained tumor tissue substantially. In addition, both a manual and semi-quantitative scoring method were used, and several different scoring systems exists. This is a subjective estimate and interobserver variability is unavoidable. Third, the small sample size of tissues from lymph node metastases and tissues from local tumor recurrence compared to primary tumors limits the interpretation of the results. This study does not provide new diagnostic methods in pathology to diagnose OSCC earlier than with current methods, but rather focuses on the potential future targets for molecular imaging.

## 4. Materials and Methods

### 4.1. Patient and Tissue Selection

From an existing, well-defined database consisting of patients diagnosed with OSCC between 2000–2011 and surgically treated at the department of Otolaryngology, Head and Neck Surgery and Audiology at Rigshospitalet (Copenhagen, Denmark), we randomly selected 41 patients. Microscopy slides were retrieved from the archives of the Department of Pathology and one FFPE tissue block containing both tumor tissue and normal epithelium were selected from each patient for following IHC staining. Of the 41 patients, 28 patients also had available tissue from lymph node metastases and 8 patients from recurrent disease. Clinicopathological data were obtained from medical and pathology reports. The 7th edition of the TNM Union for International Cancer Control (UICC) staging system was used.

### 4.2. Selection of Imaging Targets

Through literature search, we identified nine targets with previously described overexpression in several cancers, including head and neck, and for which there is a potential for rapid translation into clinical settings due to earlier research/probe development. The following biomarkers were selected: integrin αvβ6, tissue factor, poly(ADP-ribose) polymerase 1 (PARP-1), urokinase plasminogen activator receptor (uPAR), vascular endothelial growth factor receptor 1 (VEGFR1), epithelial cell adhesion molecule (EpCAM), vascular endothelial growth factor receptor 2 (VEGFR2), Cathepsin E, and integrin αvβ3. Immunohistochemical staining for cytokeratin 5 (CK5) was used to visualize tumor location. Despite its great imaging potential, epidermal growth factor receptor (EGFR) was not included as it is very well characterized in OSCC and clinical trials with targeted tracers are currently being performed (NCT03134846 and NCT03733210).

### 4.3. Immunohistochemistry

The expression of all targets was determined for both the primary tumor, metastasis, and tissue from local recurrence. Tumor tissue had been fixated in 10% formalin solution at room temperature for 24 h and then embedded in paraffin at the time of collection. FFPE blocks were stored at room temperature. Tissue sections of 4 μm were cut and IHC staining with integrin αvβ3, integrin αvβ6, tissue factor, and EPCAM were performed using a semi-automated autostainer, Ventana Benchmark Ultra (Roche Diagnostics). Manual staining was performed for the following biomarkers: Cathepsin E, PARP-1, uPAR, VEGFR1, and VEGFR2. Antibodies, reagents, and methods used for IHC analysis are listed in Appendix A. Briefly, the slides were incubated at 60 °C for 60 min before being deparaffinized in HistoClear solution, rehydrated in graded ethanol, and submerged in water. Different antigen retrieval methods were used depending on the target. All antibodies were used at optimal dilutions, which were determined using positive and negative control staining (data not shown). Secondary staining with HRP-conjugated antibody was performed by incubation for 30–40 min. The reaction was visualized with Envision DAB+ for the manual staining and with DAB+ chromogen solution for the autostainer. Digital pictures for Figure 4 were obtained using Zeiss Axioscan with 10 × zoom.

### 4.4. Assessment of Immunohistochemical Staining

Two specialized head and neck pathologists (GL and AF) reviewed and scored all samples blinded to clinical data. In the event of a disagreement, individual slides were examined together to obtain a consensus score. Each sample was assessed according to highest staining intensity in tumor compartment, proportion of stained malignant tumor tissue in the total tumor area, expression pattern in tumor tissue (homogenous or heterogeneous), and intensity in normal epithelium. Proportion and intensity scores were generated using a point system: 0% (0), 1–10% (1), 11–50% (2), 51–75% (3), and 76–100% (4), and none (0), weak (1), medium (2), and strong (3), respectively. The staining intensity of normal epithelium around the tumor tissue was scored in the same way. The proportion and intensity scores for tumor tissue were multiplied to provide a single combined score and a total immune staining score (TIS), which is similar to previous studies [38,57,58,59]. This resulted in a score ranging from 0 to 12, which was divided into four final expression categories: 0 = absent; 1–5 = low; 6–8 = intermediate; and 9–12 = high expression. For each target, the proportion of patients categorized as low, intermediate, and high expression was calculated. The expression rate was calculated as the proportion of samples with low, intermediate, and high expression.

### 4.5. Statistical Analysis

Statistical analysis was performed using IBM SPSS statistics 25.0. The median and interquartile range of the staining score were calculated for primary tumor, lymph node metastases, and recurrence. Wilcoxon signed-rank test was used to compare the intensity of immunohistochemistry staining of tumor to normal oral mucosal epithelium. Correlation between total immune staining scores in primary tumor and in lymph node metastases was tested using Spearman’s correlation test. Results were considered statistically significant at the level of *p* < 0.05.

Bar charts were made using GraphPad Prism version 9.3 for PC, GraphPad Software, La Jolla, California, USA.

## 5. Conclusions

In conclusion, the uPAR, integrin αvβ6, and tissue factor are promising imaging targets for OSCC. Molecular imaging based on a single target that could be used for both pre- and intraoperative imaging of a primary tumor, lymph node metastases, and in cases, of recurrence would be a powerful tool for the diagnosis and treatment of OSCC.

## Figures and Tables

**Table 1:** Clinicopathological characteristics of 41 patients with oral squamous cell carcinoma.

Characteristics	n (%) or Median (Range)
Age, years	58 (23–81)
Gender	
Male	26 (63%)
Female	15 (37%)
Location	
Tongue	18 (44%)
Floor of mouth	23 (56%)
Tumor differentiation	
Low	5 (12%)
Moderate	30 (73%)
High	6 (15%)
Pathologic T-stage	
T1	11(27%)
T2	17 (42%)
T3	5 (12%)
T4	5 (12%)
Missing	3 (7%)
Pathologic N-stage	
N0	3 (7%)
N+	38 (93%)

**Table 2:** Median and interquartile ranges of intensity, proportion, and total immune staining scores for each target in primary tumor, lymph node metastases, and tissue from local recurrence.

Target	Primary Tumor				Lymph Node Metastases				Local Recurrence				Normal Epithelium	
	n	Intensity score (IQR)	Proportion (IQR)	TIS-score (IQR)	n	Intensity score (IQR)	Proportion (IQR)	TIS-score (IQR)	n	Intensity score (IQR)	Proportion (IQR)	TIS-score (IQR)	n	Intensity score (IQR)
Integrin αvβ6	40	3(3-3)	4(4-4)	12(12-12)	28	3(3-3)	4(4-4)	12(9.75-12)	8	3(3-3)	4(4-4)	12(12-12)	47	3(3-3)
Tissue factor	41	3(2-3)	2(1-3)	6(2.5-7.5)	28	2(1-3)	1(1-2)	2(1-5.5)	8	2.5(0-3)	1.5(0-2)	4(0-6)	47	1(1-1)
PARP1	35	2(2-3)	3(3-4)	6(4-9)	24	2(2-3)	3(3-3)	6(6-9)	7	2(2-2)	3(3-4)	6(4-8)	39	2(1-2)
uPAR	34	3(2.75-3)	2(2-3)	6(6-9)	23	3(2-3)	2(2-3)	6(4-6)	6	3(2.75-3)	2.5(2-3.25)	6(6-9.75)	37	0(0-0)
VEGFR1	35	2(2-2)	4(3-4)	8(6-8)	24	2(2-2)	4(3.25-4)	8(6.5-8)	7	2(1-2)	4(3-4)	8(3-8)	40	1.5(1-2)
EpCAM	34	0.5(0-2)	0.5(0-2)	0.5(0-2.5)	24	1.5(0-3)	1(0-1)	1.5(0-3)	7	1(0-3)	1(0-2)	1(0-6)	34	0(0-0)
VEGFR2	35	1(1-2)	1(1-2)	2(1-4)	24	1(1-2)	2(1-2)	2(1-4)	7	1(0-1)	2(0-2)	2(0-2)	39	0(0-1)
Cathepsin E	35	0(0-0)	0(0-0)	0(0-0)	23	0(0-0)	0(0-0)	0(0-0)	7	0(0-0)	0(0-0)	0(0-0)	40	0(0-0.5)
Integrin αvβ3	36	0(0-0)	0(0-0)	0(0-0)	27	0(0-0)	0(0-0)	0(0-0)	8	0(0-0)	0(0-0)	0(0-0)	44	0(0-0)

**Table 3:** Overview of the expression pattern for all nine included targets.

Target	Tumor-Specific	Homogenous Expression in Tumor Compartment	Expression Rate Primary Tumor	Expression Rate Lymph Node Metastasis	Superficial Margin Contrast Tumor vs. Epithelium	Profound Margin Contrast Tumor vs. Normal Cells
Integrin αvβ6	Partly	80%	95%	100%	No	Yes
Tissue factor	Yes	3%	93%	80%	Yes	Yes
PARP1	No	0%	94%	100%	Yes	No
uPAR	Yes	51%	97%	96%	Yes	Yes
VEGFR1	No	5%	100%	100%	Yes	No
EpCAM	No	3%	50%	66%	No	No
VEGFR2	No	0%	80%	81%	Yes	No
Cathepsin E	NA	0%	3%	13%	NA	NA
Integrin αvβ3	NA	NA	NA	NA	NA	NA

## Data Availability

The data reported in this study are available from the corresponding authors upon reasonable request.

## Appendix A

**Table A1:** Primary antibodies used for immunohistochemistry.

Target	Source	Catalog Number	Species	Monoclonal/Polyclonal	Dilution	Staining Method
Cytokeratin 5	Novocastra	CK5-L-CE	Mouse	Monoclonal	No dilution	Automatic, VENTANA BenchMark IHC
Integrin αvβ3	R&D Systems	MAB3050	Mouse	Monoclonal	1:500	Automatic, VENTANA BenchMark IHC
Integrin αvβ6	ABCAM	ab181551	Mouse	Monoclonal	1:500	Automatic, VENTANA BenchMark IHC
Tissue Factor	Sekisui	4509	Mouse	Monoclonal	1:50	Automatic, VENTANA BenchMark IHC
EPCAM	Cell Marque	5435676001	Mouse	Monoclonal	No dilution	Automatic, VENTANA BenchMark IHC
Cathepsin E	Abcam	Ab36996	Rabbit	Polyclonal	1:2000	Manual
PARP1	Abcam	Ab32138	Rabbit	Monoclonal	1:25	Manual
uPAR	Genetex	GTX100467	Mouse	Monoclonal	1:500	Manual
VEGFR1	Abcam	Ab32152	Rabbit	Monoclonal	1:75	Manual
VEGFR2	Cell Signaling	55B11	Rabbit	Monoclonal	1:300	Manual
